# Rectal ulcers: a complication from stool management system use

**DOI:** 10.1093/omcr/omad040

**Published:** 2023-05-30

**Authors:** Muhammad Haider, Madiha Master, Muhammad Master, Evelina Khalilova, Magda Daoud, Jay Nfonoyim

**Affiliations:** Department of Medicine, Richmond University Medical Center/Mount Sinai, Staten Island NY, USA; Philadelphia College of Osteopathic Medicine, Philadelphia PA, USA; University of North Texas Health Science Center, Fort Worth TX, USA; Department of Medicine, Richmond University Medical Center/Mount Sinai, Staten Island NY, USA; Department of Gastroenterology, Richmond University Medical Center/Mount Sinai, Staten Island NY, USA; Department of Medicine, Richmond University Medical Center/Mount Sinai, Staten Island NY, USA

## Abstract

Fecal management System (FMS) is widely used across medical facilities in United States. These devices have helped in preventing problems associated with fecal incontinence. Although highly efficient, these devices can also lead to certain complications including rectal ulcer and lower Gastrointestinal (GI) bleed. Here, we report a case of a 56-year-old male being treated for pneumonia and atrial fibrillation, who had significant lower GI bleed while being on FMS for stool incontinence. A colonoscopy was performed, which showed multiple rectal ulcers with one large ulcer having a visible pulsating vessel. This case highlights a rarely reported complication of rectal ulcers and GI bleed associated with use of FMS.

## INTRODUCTION

Fecal incontinence remains a common and difficult problem for hospitalized patients. It is reported in ⁓50% of patients admitted to hospitals and long care facilities [1]. It can occur in both sexes and patients of all ages. Managing critically ill patients with diarrhea or fecal incontinence is a challenge as it can lead to infections, skin breakdown and ulcerations [2] Several solutions for fecal incontinence existed including medications such as loperamide and opiate, products such as absorbent pads, rectal foley catheters, artificial sphincters, even colostomy [3]. In the past decade, several fecal management systems (FMSs) have been introduced to deal with diarrhea and incontinence, especially in immobile and critically ill patients. These systems have helped in reducing perianal skin excoriation, infection spread and the overall nursing requirements. Despite the effectiveness and a high safety profile of these devices, there have been reports of adverse events in the published literature. These complications result mostly from pressure injury or traumatic device removal; and may include rectal ulcer, fissures and major traumatic rectal bleed. Here, we present a case of 56-year-old male who had major lower Gastrointestinal (GI) bleed as result of rectal ulcers from FMS use.

## CASE PRESENTATION

A 56-year-old male with extensive past medical history including diabetes mellitus, hypertension, congestive heart failure, chronic obstructive pulmonary disease (COPD), intracranial bleed from aneurysm rupture, on hemodialysis for end stage renal disease, who presented with complain of shortness of breath. Patient was started on treatment for pneumonia and COPD exacerbation. While in the hospital the patient became obtunded and was intubated for airway protection. Patient was upgraded to medical intensive care unit (MICU) for further management.

While in the MICU patient developed new onset atrial fibrillation and was started on a heparin drip. Patient also was found to have fecal incontinence and a Dignishield FMS was placed. In 2 days after placement of rectal tube, patient had an episode of hematochezia that was small in amount. Heparin was put on hold and hemoglobin was closely monitored. Three days later patient had another large episode of hematochezia, with ⁓150 mL of blood loss and visible clots resulting in hemodynamic instability. Code fusion was initiated and patient was adequately resuscitated with blood transfusion and intravenous fluids. A computed tomographic angiography didn’t reveal any active bleed in the GI tract. Gastroenterology service was consulted who performed an urgent upper endoscopy to rule out any upper GI bleed. The upper endoscopy failed to reveal any bleeding source.

**Figure 1:**
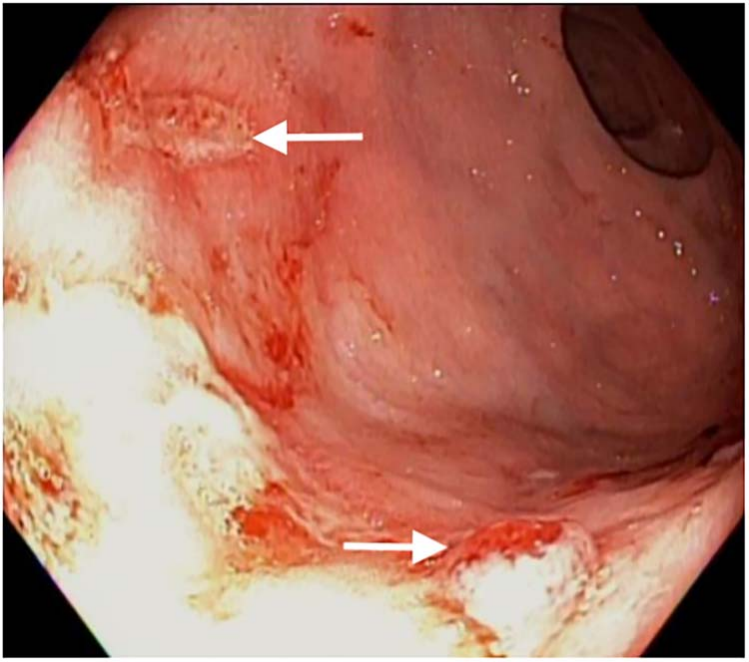
White arrows shows multiple rectal ulcers.

**Figure 2:**
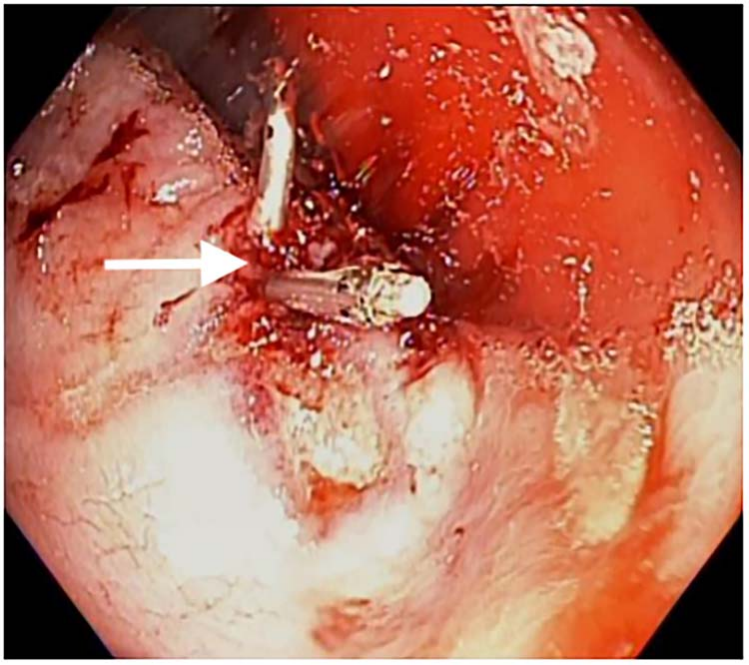
White arrow shows ulcer closure with endoclips.

Patient then had bowel prep with polyethylene glycol and underwent colonoscopy the next day. Colonoscopy revealed multiple superficial rectal ulcerations varying in size (Fig. 1). It also showed one large deep ulcer, 2 cm in size, was seen in the rectum with a pulsating visible vessel in ulcer base (Fig. 2), 3 mL of diluted epinephrine (1:10 000) was injected in ulcer base followed by application of three endoclips to the ulcer with adequate hemostasis. This patient had a previous screening colonoscopy 1 year prior that was negative for any rectal ulcers or inflammatory bowel disease.

The rectal tube was discontinued after these findings. Patient was also started on daily polyethylene glycol to avoid constipation. He completed his course of treatment without any further bleeding and was eventually discharged from the hospital.

## DISCUSSION

Fecal incontinence is the involuntary recurrent passage of stool. Its more commonly seen in patients with advance age, multiparous women, history of obstetric complications and those with anorectal disease or surgery. The availability of FMS provides effective management for fecal incontinence in patients of both short and long care facilities.

Currently there are three main FMSs available in the United States; these includes Flexi-Seal FMS (ConvaTec); Bowel Management System (Hollister) and DigniCare Stool Management System (Bard). All these FMS have a catheter with an inflatable balloon on one end, which keeps the catheter from being dislodged from the rectum; on the other end of the catheter is an external collection bag for stool collection.

There are potential complications associated with the use of a FMS, these can include loss of anal sphincter muscle tone, infection, abdominal distention, bowel obstruction and perforation, ulcers and rectal bleeding [4]. Our Patient also developed rectal ulcer with severe GI bleed. The mechanism by which these ulcers develop is due to pressure injury caused by the inflatable balloon. In addition, the buildup of stool above the catheter may also contribute to pressure on the bowel wall resulting in necrosis. This follows similar mechanism as seen in stercoral perforation due large stool burden in severely constipated patients. [5]. Both major brands i.e. Flexi-Seal FMS (ConvaTec) and DigniCare Stool Management System (Bard) recommend inflating the cuff with 45 mL of tap water and to discontinue use after 29 days. All the patient reported in literature developed bleeding complications between day 3 to 3 weeks post insertion [6, 7]. Review of literature shows ⁓14 cases reporting rectal bleed in patients on these stool management systems. In five of these case [6, 8–11] the patients were on therapeutic dose of heparin, warfarin or direct oral anticoagulants. This was also the case in our patient who was on therapeutic dose heparin for atrial fibrillation. Although no studies are available that discuss association between anticoagulation use and ulcer bleed in these patients, both the cases reported in the literature and our case, should keep physicians cautious about severe bleed from rectal ulcer in patients using FMS while on anticoagulation.

## CONCLUSION

Our case highlights the fact that FMS although safe can have severe complications. Ulcer formation occurs as a result of pressure injury from these devices that employ an inflatable cuff mechanism. Also, physicians need to be cautious when using these devices in patient on anticoagulation and should reassess the need for these catheters every few days.

## DATE AVAILABILITY

The corresponding author may be reached out for clinical data.
